# Effect of BRAF^V600E ^mutation on transcription and post-transcriptional regulation in a papillary thyroid carcinoma model

**DOI:** 10.1186/1476-4598-6-21

**Published:** 2007-03-13

**Authors:** Susanne Cahill, Paul Smyth, Karen Denning, Richard Flavin, Jinghuan Li, Astrid Potratz, Simone M Guenther, Richard Henfrey, John J O'Leary, Orla Sheils

**Affiliations:** 1 Dept. of Histopathology, University of Dublin, Trinity College, Dublin, Ireland; 2 Applied Biosystems, Foster City, CA, USA

## Abstract

**Background:**

microRNAs (miRNAs) are a group of non-coding single stranded RNAs measuring approximately 22 nucleotides in length that have been found to control cell growth, differentiation and apoptosis. They negatively regulate target genes and have recently been implicated in tumourigenesis. Furthermore, miRNA expression profiling correlates with various cancers, with these genes thought to act as both tumour suppressors and oncogenes. Recently, a point mutation in the BRAF gene leading to a V600E substitution has been identified as the most common genetic change in papillary thyroid carcinoma (PTC) occurring in 29–69% of cases. This mutation leads to aberrant MAPK activation that is implicated in tumourigenesis.

**Aim:**

The aim of this study was to identify the effect that BRAF oncogene has on post-transcriptional regulation in PTC by using microRNA analysis.

**Results:**

A unique miRNA expression signature differentiated between PTC cell lines with BRAF mutations and a normal thyroid cell line. 15 miRNAs were found to be upregulated and 23 miRNAs were downregulated. Several of these up/down regulated miRNAs may be involved in PTC pathogenesis. miRNA profiling will assist in the elucidation of disease pathogenesis and identification biomarkers and targets.

## Background

Papillary thyroid carcinoma (PTC) is the most commonly occurring thyroid malignancy accounting for approximately 80% of cases. Genetically PTC is defined by several alterations which cause abnormal activation of the mitogen-activated protein kinase (MAPK) pathway, the most prevalent being point mutations in the intracellular signalling kinase BRAF [1,2]. An activating mutation in BRAF (V600E) leads to constitutive activation and hence de-regulation of MAPK pathway. Several groups have reported that PTCs harbouring mutations in BRAF have more aggressive properties, present more often with extra-thyroidal invasion and also at a more advanced stage. Histological features such as the classic PTC architecture and tall cell features are also associated with the BRAF oncogene [3,4].

Despite this range of knowledge thyroid carcinoma still poses a significant diagnostic challenge and understanding of the complete range of genes and pathways involved in its pathogenesis is incomplete. Until recently biomarker discovery has concentrated largely on protein coding RNAs in a quest to identify cancer associated genes involved in thyroid carcinogenesis [5-7]. The recent discovery however that a large group of non-coding RNAs called microRNAs (miRNAs) may potentially play a role in cancer has led to a proliferation of miRNA related research of late including the publication of several key reviews [8-12]. These small non-coding RNAs constitute a novel class of gene regulators that function by negatively regulating gene expression by targeting mRNAs for cleavage or translational repression. Several recent studies on the regulatory functions of miRNAs suggest that they play critical roles in central processes such as development, cell proliferation and differentiation, stress resistance, metabolism and apoptosis all of which are involved in tumourigenesis [13,15]. Not surprisingly aberrant miRNA expression has been implicated in cancer development and a hypothesis has emerged suggesting that tumours may each have a discrete "miRNA signature" [16-20]. Differential expression of miRNAs between malignant tissue and normal tissue and between different types of tumour, has been shown in several key studies, indicating that miRNAs are determinants of clinical diagnostic and prognostic significance [21,22].

Recently several groups have utilised a range of techniques most notably northern blot analysis, cloning and oligonucleotide microarrays, in order to analyse miRNA expression profiles in different cancer types [23-26]. In this study a novel stem-looped TaqMan^® ^RT-PCR method was used to measure the expression levels of a panel of 160 mature miRNAs in thyroid cell lines containing BRAF mutation.

## Results

### Differential miRNA expression using TaqMan™ microRNA assays

Stem-looped TaqMan^® ^RT-PCR was used to measure the expression levels of 160 miRNAs in a collection of cell lines including a normal thyroid cell lines N-thy-ori 3-1 and two cell lines containing V600E BRAF point mutation [Nthy-oriBRAF and KAT-10]. A comparison was then made between Nthy-ori and BRAF mutated cell lines to determine miRNAs whose expression was differentially expressed between the two groups. ΔΔC_T _method was used to calculate fold change of miRNA expression between groups. 15 miRNAs were found to be upregulated with a fold change of > 2 in BRAF mutated cell lines when compared to normal.

Three of these upregulated miRNAs showed significantly higher fold change than the other upregulated miRNAs, these are mir-200a, mir-200b and mir-141.

**Figure 1:**
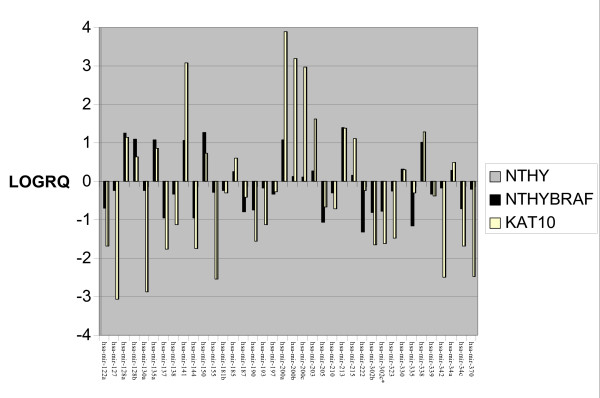
**Differentially expressed miRNAs Between BRAF V600E harbouring Cell Lines and Normal Thyroid Cell line**. Delta delta CT was performed using Nthy-ori 3-1 as a normal control. The log of the RQ values was used to plot the relative fold change of Nthy-BRAF and KAT10 against Nthy-ori 3-1. miRNAs are on the × axis

**Figure 2:**
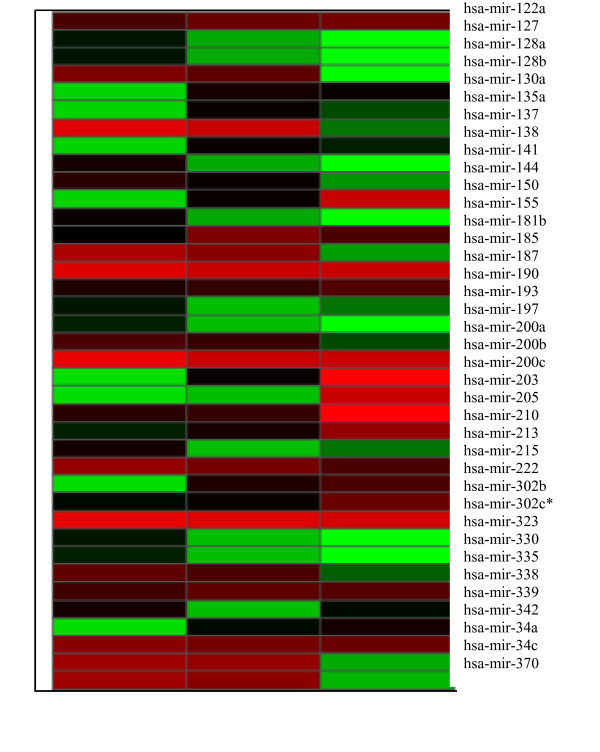
**Heat map of MiRNA expression**. miRNA expression. Each miRNA listed was detected as significantly differentially expressed between BRAF mutated cell lines and N-thy-ori. The delta CT values for each miRNA were used to create the heat map

23 miRNAs showed underexpression of > 2 fold when compared to the normal thyroid cell line. Moreover, three miRNAs showed significantly more underexpression compared to the other downregulated miRNAs. These miRNAs are as follows mir-127, mir-130a and mir-144. (Table 1 and Figures 1 and 2).

**Table 1:** Differentially expressed miRNAs in BRAF mutated cell lines V normal

**Upregulated**	**Downregulated**
has-mir-128a	hsa-mir-122a
has-mir-128b	hsa-mir-127
has-mir-135a	hsa-mir-130a
has-mir-141	hsa-mir-137
has-mir-150	hsa-mir-138
has-mir-185	hsa-mir-144
has-mir-200a	hsa-mir-155
has-mir-200b	hsa-mir-181b
has-mir-200c	hsa-mir-187
has-mir-203	hsa-mir-190
has-mir-213	hsa-mir-193
has-mir-215	hsa-mir-197
has-mir-330	hsa-mir-222
has-mir-338	hsa-mir-302b
has-mir-34a	hsa-mir-302c
	hsa-mir-323
	hsa-mir-335
	hsa-mir-339
	hsa-mir-342
	hsa-mir-34c
	hsa-mir-370

### mRNA (transcription) and miRNA (regulation) expression correlation

miRNAs interact and regulate their target genes by cleavage or translational inhibition [12]. A current challenge is to identify biologically relevant targets that are regulated by individual miRNAs. This task is further complicated by the fact that each miRNA can potentially bind and regulate many mRNA targets and each mRNA can be bound and regulated by several miRNAs. Several publicly available databases exist for prediction of miRNA targets. MiRBase, PICTAR and TARGETSCAN were used in this study [26-28].

These three databases were utilized to yield lists of putative targets for each miRNA which are illustrated in table 2. From these lists there are several genes that are biologically relevant in the context of PTC. The miRNAs which showed the highest increase in fold change are predicted to bind genes involved in thyroid function, MAPKKK cascade, retinoic acid receptors, cell adhesion and cell structure associated genes and genes involved in cell signalling. miRNAs which showed the largest decrease in fold change are predicted to regulate and target genes involved in G-protein mediated signalling, oncogenesis and cell cycle control. One might predict that a miRNA which is overexpressed would be associated with downstream underexpression of its target. This regulation can occur either at the transcript level or the protein level.

**Table 2:** Predicted targets for differentially expressed miRNAs

**miR-200a**	IRS2, MAP3KA, MYH10, SLC20A1, YWHAG, E2F3, RAB38, PPP2R2A, CALCR, MTSS1, RAB30, STXBP1, THRAP2, STRN, MATR3, MTPN, FGFR2
**miR-128b**	PLK2, PRKD1, ADORA2B, ZNF385, CASC3, PAIP2, ITGB4BP CRKL, CDH24, ING5, BAG2, EGFR, ST14, THRAP1 CORO1CE2F, E2F3, PDGFRA, INSR, CITED2, STX16
**miR-141**	PDCD4, MYH10, WTAP, STAT5A, PERP, TCF8, FUS, MYL4 LBR, SOCS6, ACTA,1MLANA, CDK2, MAGED4, EIF3S9, HISTIH2AG KRT10, XPO4, DCTN3
**miR-127**	RIMS4, TLK2, BCL6, TFF1, VASH1, PCDH21, BAD, TTGB5, ICAM 3, TMEMG, HIST4H4, APOB, LGALS8, CCNK,
**miR-34c**	RAB43, CELSR3, NOTCH1, MET, CALCR, NOTCH2, THRAP2, TPO52, DAAMI, PDGFRA, MAD1A
**miR-302c**	RAB43, CELSR3, NOTCH1, MET, CALCR, NOTCH2, THRAP2, TPO52, DAAMI, PDGFRA, MAD1A

miR-200a, miR-128b, miR-141 = Upregulated miRNA

miR-127, miR-34c, miR-302c = Downregulated miRNA

## Discussion

microRNAs (miRNAs) are now recognized as an additional layer of post-transcriptional control that must be considered in order to understand the complexity of gene expression in humans. miRNAs are now known as a novel class of gene regulators that are involved in cancer related processes and deregulation or aberrant expression of miRNAs may contribute to human diseases, including cancer. Calin et al showed down-regulation of miRNA-15 and miRNA-16 in a majority of chronic lymphatic leukaemia (CLL). Altered miRNA expression has also been reported in breast cancer, glioblastoma, lung cancer and colorectal cancer [29-35].

This study utilized a novel stem-looped TaqMan RT-PCR method to measure the expression levels of 160 mature miRNAs in a collection of thyroid cell lines. This method offers several distinct advantages over conventional miRNA detection methods including a higher sensitivity and specificity and a fast and simple methodology. The aim of this study was to correlate BRAF mutant status with an associated miRNA profile, with a view to elaborating the downstream regulatory effect of BRAF mutation.

The data showed a set of differentially expressed miRNAs in cell lines with BRAF V600E mutation compared to a normal thyroid cell line (Table 1). There were 15 overexpressed miRNAs and 23 underexpressed miRNAs in these cell lines compared to the normal cell line. Of these miRNAs listed in table 1 only a few have been previously associated with other cancers. One of which is miR-127, that showed down-regulation in BRAF V600E mutated cell lines. Human miR-127 is embedded in a CpG island and is silenced in several human cancers including bladder, prostate and colon cancer and has been suggested to play a role as a possible tumour suppressor gene [33]. miR-127 also an example of the potential utility of miRNA targeted therapy in cancer. Saito et al demonstrated that induction of miR-127 by with 5-aza-20-deoxycytidine and 4-phenylbutyric acid, which inhibits DNA methylation and histone deacetylase respectively, reduces expression of the oncogene BCL6 in bladder cancers [33]. Its utility as a potential cancer target has yet to be explored in other cancers however this suggests the possibility of direct targeting of miRNAs that are amplified or upregulated in patient tumours. Two other miRNAs listed in table 2 have also been described in other cancers. These include miR-200b and miR-141 which have been shown to be highly overexpressed in malignant cholangiocytes and in colon carcinoma. [18]. This suggests the possibility of miR-200b and miR-141 down-regulating possible tumour suppressor genes. Of particular interest in this study is the downregulation of miR-323 and miR-302b in BRAF mutated cell line. miRBASE target database predicted that the BRAF transcript has binding sites for miR-323 and miR-302b to potentially bind and down- regulate BRAF expression.

A number of miRNAs exhibited a difference in expression level between KAT10 cell line and normal but did not show a significant change in the BRAF transfected cell lines and normal. miRNAs specific to the PTC cell line with native mutant BRAF included, mir-10a [overexpressed with a fold change of 95 in KAT10] mir-23b [downregulated with a fold change of 2949] and mir-154 [downregulated with a fold change of 142]. It is possible these miRNAs may play a role in a later stage in tumour formation and progression; given the transfected cell line had only been exposed to mutant BRAF for a short period (3 passages) unlike KAT10 which represents a genuine PTC which has evolved with BRAT mutant as an intrinsic component. Alternatively, the difference may reflect the accumulation of several genetic insults in the process of tumourigenesis.

Recent studies in miRNA deregulation PTC found an aberrant miRNA expression profile in PTCs compared to normal thyroid tissues [36,37]. In particular, a significant increase in mir-222, mir-221, mir-146. Our study did not find any of these miRNAs to be significantly overexpressed in the cell lines examined. miR-222 was actually found to be downregulated in the BRAF mutated cell lines. Another miRNA these two groups found to be upregulated albeit less significantly was miR-213, our study also found miR-213 to be upregulated in BRAF mutated cell lines [25 fold]. These differences may be due to the different experimental approaches; Huiling et al and Pallante et al used miRNA microarrays. They also used different chemistry and fresh tissue samples; also the *RET *and *BRAF *status of each PTC case was not disclosed.

Prediction of miRNA targets is an immediate challenge in miRNA research. Several computational approaches have been implemented for miRNA gene prediction using methods based on sequence conservation and/or structural similarity. To overcome the limitations of target prediction three of these databases MiRBase, TARGETSCAN and PICTAR were used in this study to predict possible targets of the differentially expressed miRNAs associated with BRAF mutated (Table 2). Examples of other programs developed for target identification include RNAhybrid, RNA calibrate and RNAeffective. Only a few target mRNAs have been experimentally proven and studied *in vitro *and not all the prediction algorithms yield overlapping lists therefore the lists may contain several false positives that may fit the criteria but do not interact with the miRNA *in vitro*. To overcome this, results were intersected from these databases to identify the genes commonly predicted by at least two of the algorithms. The results of the potential targets of several differentially expressed miRNAs are displayed in table 2.

It is reasonable to expect that targets of downregulated miRNAs include oncogenes or genes encoding proteins with potential oncogenic function. Indeed among putative targets, several genes with potential oncogenic functions were found include RAB35, ERBB4, MAP3K3, VASP6, KIT, FOSB, NOTCH1. Upregulated miRNAs are purported to interact with tumour suppressor genes. Those identified from this study include MAD 4 and ST6. Several extracellular membrane molecules were also identified including; myotubulin, MAD1A, Cadherins. In general overexpression of an miRNA would be associated with downregulation of its target miRNA.

Interestingly among these potential gene targets are a number of genes that have implications in thyroid carcinoma progression such as genes involved in thyroid function, cell cycle control, cell signalling, MAPKKK cascade, retinoic acid receptors, cell adhesion and cell structure associated genes. Therefore, it seems plausible that this subset of miRNAs may be important in the progression of thyroid carcinoma by working together or independently to regulate genes involved in thyroid tumourigenesis

**Figure 3:**
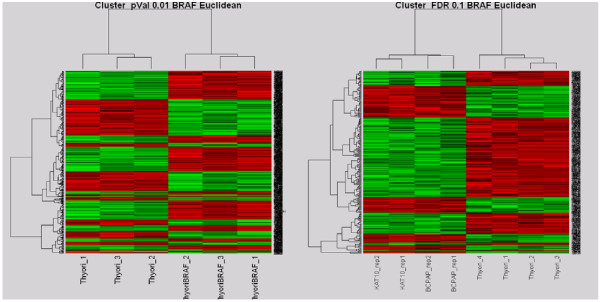
**Hierarchical Clustering of BRAF mutated Cell lines**. In (a) The column dendrogram clearly shows cases clustering on the basis of BRAF mutation. To the left are the normal thyroid cell lines with no BRAF mutation and to the right are Nthy-BRAF with V600E mutation. In (b) The denodrogran shows cases again clustering on the basis of BRAF mutation. To the left are the PTCs with BRAF V600E mutation and to the right are the normal thyroid cell lines with no mutation.

Our group has also previously examined gene expression levels in these same cell lines. This analysis was done by evaluating genome-wide gene expression levels in BRAF mutated cell lines and matched normal thyroid cell lines using the Applied Biosystems Gene Expression Arrays. Treatment comparisons were preformed resulting in lists of differentially expressed genes (Figure 3). These gene lists were uploaded into PANTHER (Protein ANalysis THrough Evolutionary Relationships) classification system and the software statistically compared them to a reference list to look for under and over-represented molecular functions and biological process. Analysis revealed a set of genes associated with mutant BRAF expression which are involved in processes such as apoptosis, chromosomal instability, invasiveness and metastasis and proliferation. Other genes identified were shown to be involved in the insulin-like growth factor pathway (including (insulin-like growth factor binding protein) IGFBP4, (insulin receptor substrate) IRSI, GRB14, PLAG1 and IGF2) and the Wnt signaling pathway suggesting their importance in BRAF signalling.

Up and down regulation of cell adhesion and chromosome remodelling molecules were also shown. BRAF mutation has previously been shown to induce chromosome instability and decrease cell- cell interactions [38] and our data further corroborates the involvement of these processes in papillary thyroid carcinoma. Not surprisingly genes involved in the MAPK pathway, of which BRAF is an integral part, showed an increase in expression. Among these genes were DDIT, DUSP6, TFPI-2, Seladin-1, MAP3K12, and GRB14. These results provided an insight into the different pathways through which BRAF exerts its oncogenic effect and emphasised the importance of the MAPK and Wnt signalling pathways in thyroid tumour formation.

The list of putative miRNA targets (as defined above in table table 1) correlated with the list of differentially expressed genes on analysis. This is illustrated in table 3. It is interesting to speculate that these genes are regulated by their corresponding miRNA thereby contributing to tumour development in thyroid. These observations should be placed in context, in so far as predictive databases are incomplete and target selection is essentially speculative and involves a degree of subjectivity as these target mRNAs have not been experimentally proven and studied *in vitro*.

**Table 3:** miRNAs and Predicted Targets.

**Up-regulated Genes**	**Potential Binding Partner (down-regulated)**
PGM5	miR-137
DUSP6	miR-138
TLE	miR-323
HSPH1	miR-181b
PCGF1	miR-138
CDK2AP2	miR-302c, miR-302b
DDIT3	miR-144
SLC14A1	miR-222
MANEA	miR-302b, miR-302c
ELP4	miR-122a
APIP	miR-181b
PEX7	miR-190

**Down-regulated Genes**	**Potential Binding Partner (up-regulated)**

SYTL2	miR-128a, miR-128b
GJB3	miR-34a
SDC4	miR-141, miR-215
CREBL2	miR-141, miR-200a,
CCL2	miR-141, miR-200a
GYG2	miR-215
HIATL1	miR-34a
ZMYND11	miR-200a
ADAMTS4	miR-141

Differentially expressed miRNAs in BRAF mutated cell lines. Red = Upregulated. Green = Downregulated

In summary this study revealed a set of miRNAs associated with expression of mutant BRAF^V600E ^oncogene in PTC cell lines when compared to a normal thyroid cell line. The BRAF oncogene may influence miRNA expression directly or indirectly by altering the expression patterns of genes, such as transcription factors, involved in tumour progression or indeed the genes involved in the biogenesis and synthesis of miRNAs such as those from the RISC complex and miRNA machinery. These miRNAs associated with BRAF oncogene as listed in Table 1 may contribute to tumour formation by regulating genes involved in the MAPK pathway in which BRAF plays a role.

This data suggests the potential utility of miRNA expression patterns as a molecular screen for PTC to use as a diagnostic adjuvant to already established morphological criteria. A recent study by this group examined the effect the ret/PTC 1 oncogene has on miRNA expression in PTC cell lines. ret/PTC 1 rearrangements are generally associated with classic papillary architecture and indolent behaviour in thyroid carcinoma. This study showed a distinct pattern of miRNAs associated with the ret/PTC 1 oncogene when compared to a normal thyroid cell lines [39].

It is noteworthy that different miRNA expression profiles have been shown contingent upon ret/PTC 1 activated and BRAF mutated cell lines. This suggests that miRNA expression analysis may also be of prognostic significance. Further miRNA analysis in a larger cohort of samples will help validate and consolidate the results discussed above. Table 4 illustrates a compilation of miRNAs that are differentially expressed in PTC cell lines (either ret/PTC-1 or BRAF mutant), suggesting an important role for these miRNAs in thyroid neoplasia progression independent of molecular trigger. They may be involved in regulating the central MAPK pathway involved in thyroid tumourigenesis in which RET and BRAF oncogenes play a part.

**Table 4:** Differentially Expressed miRNA in ret/PTC 1 and BRAF mutated cell lines

**Upregulated miRNAs**	**Downregulated miRNAs**
miR-128a	miR-127
miR-128b	miR-302b
miR-185	miR-302c
miR-200a	miR-323
miR-200b	miR-370
miR-213	

## Conclusion

Although the exact function of many miRNAs remains to be elucidated it is clear that miRNAs potentially have a broad influence over several diverse genetic pathways and that their deregulation is likely to contribute to disease including cancer. An exciting future prospect is that the miRNA patterns associated with a particular tumour may ultimately be of diagnostic and prognostic significance and contribute to the understanding of the molecular pathogenesis and gene regulatory processes of cancer.

## Materials and methods

### Cell culture and transfection

Nthy-ori 3-1 (ECACC, Wiltshire, UK) is a thyroid follicular epithelial cell line derived from normal thyroid tissue of an adult that has been transfected with a plasmid encoding for the SV40 large T gene. KAT10 is derived from a papillary thyroid carcinoma cell line with heterozygous BRAF V600E mutation. Both cell lines were grown to confluence in a humidified atmosphere containing 5% CO_2 _at 37°C in the following plating medium: RPMI 1640 with 2 mM L-glutamine, 10% Foetal calf serum (FCS), Penicillin (100 U/ml) and Streptomycin (100 μg/ml). A BRAF V600E expressing plasmid, pMCEF-V600EB-RAF, was transfected into Nthy-ori 3-1. Transfections were carried out using Genejuice™ transfection agent (Novagen, Germany) using the recommended protocol and transfected cells were grown in the presence of Gentamicin (Sigma Aldrich) antibiotic to yield pure cultures.

### Nucleic acid extraction

**Figure 4:**
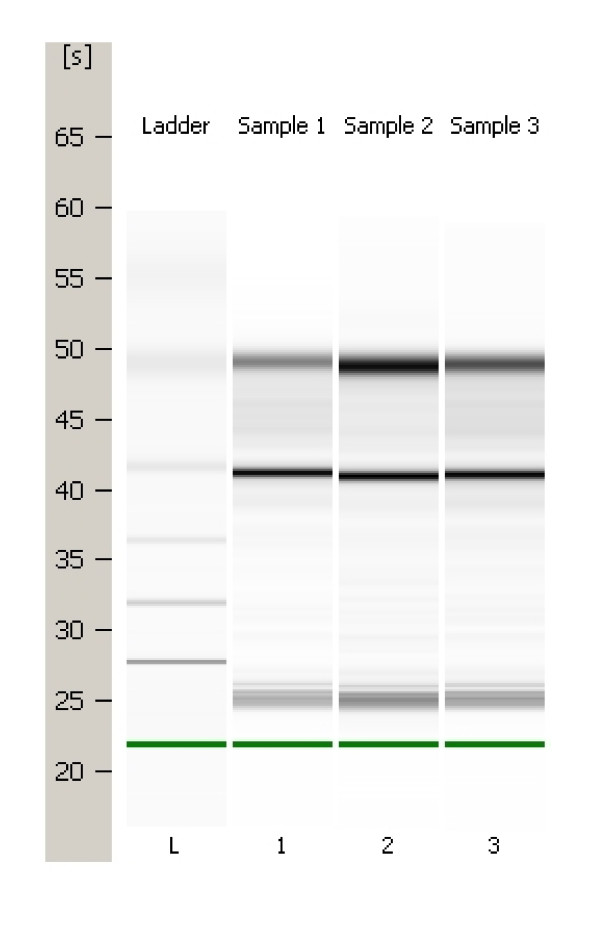
**Bioanalyser "virtual" gel electrophoresis for N-thy-ori Cell Lines**. Lane 1: Nthy-ori rep1; Lane 2:Nthy-ori rep 2; Lane 3: Nthyori rep3

Following trypsinisation of cultured cells, cell pellets (3–4 × 10^6 ^cells) were collected and total RNA was extracted using RNeasy^® ^mini kit (Qiagen Ltd., West Sussex, UK). RNA quantity and quality are assessed using a NanoDrop^® ^ND-1000 Spectrophotometer (Wilmington, USA) and RNA 6000 Nano LabChip^® ^Kit in conjunction with the Agilent 2100 Bioanalyser (Agilent technologies, Waldbronn, Germany) as illustrated in figure 4. Taqman^® ^SNP detection was used for BRAF V600E mutation detection as previously described by Smyth et al [40]. Primers and probes used in this experiment were designed and used to the manufacturer's recommendations. The primers/probes used were as follows: 5' CAT GAA GAC CTC ACA GTA AAA ATA GGT GAT 3' [BRAF-F], 5' GGA TCC AGA CAA CTG TTC AAA CTG A 3' [BRAF-R], VIC-5' CCA TCG AGA TTT CAC TGT AG 3' [BRAF-P^WT^], and FAM-5' CCA TCG AGA TTT CTC TGT AG 3' [BRAF-P^MUT^]. Amplification and analysis was performed using an ABI Prism 7000 Sequence Detection System (Applied Biosystems, CA, USA) for 40 cycles (92°C for 15 sec, 60°C for 1 min).

Western blot analysis was carried out on the transfected cell lines to confirm BRAF protein expression.

### Microarray analysis

The Applied Biosystems 1700 Expression Array System is based on a microarray design that represents the whole human genome. The V.2 array has 32,878 60-mer oligonucleotide probes for the interrogation of 29,098 individual human gene and more than 1,000 control probes. V2 arrays were used to analyse the transcriptional profiles of the cell line RNA samples in this study. Digoxigenin-UTP labeled cRNA was generated and linearly amplified from 2 μg of total RNA using Applied Biosystems Chemiluminescent RT-IVT Labelling Kit v 2.0 following the manufacturer's protocol. Array hybridization, chemiluminescence detection, image acquisition and analysis were performed using Applied Biosystems Chemiluminescence Detection Kit and 1700 Chemiluminescent Mircoarray Analyzer following manufacturer's guidelines. Each microarray was initially pre-hybridised at 55°C for 1 hr in hybridization buffer with blocking reagent. 10 μg of labeled cRNA targets were fragemented by incubating with fragmentation buffer at 60°C for 30 min, mixed with internal control target (ICT, 24-mer oligo labeled with LIZ fluorescent dye) and hybridized to each pre-hybed microarray in a 1.5 ml volume at 55°C for 16 hr. After hybridization, the arrays were washed with hybridization wash buffer and chemiluminscence rinse buffer. Enhanced chemiluminescenct signals were generated by incubating arrays with anti-digoxigenin alkaline phosphatase, enhanced with Chemiluminescence Enhancing Solution and finally by adding Chemiluminescence Substrate. Images were collected for each microarray using the 1700 analyser. Images were autogridded and the chemiluminescent signals were quantified, corrected for background and spot and spatially normalized. Replicates were performed. Microarrays were analysed using Spotfire Decision Site™ for functional genomics (Spotfire AB, Göteborg, Sweden) and R version 1.9.1 [a free language and environment for statistical analysis and graphics] (R Development Core Team, 2004).

### miRNA analysis

Applied Biosystems TaqMan^® ^microRNA (miRNA) assays are designed to detect and quantify mature miRNAs using a looped-primer real time PCR. The human early access panel used in this study contained 160 individual assays for identified human miRNAs. The assay involved 2 steps: Step one; a Stem-looped RT, and Step two; a Real Time PCR. Briefly, single stranded cDNA was generated from total RNA sample by reverse transcription using the Applied Biosystems High-Capacity cDNA Archive Kit (Applied Biosystems, CA, USA) following manufacturer's protocol. RT reactions contained 10 ng of total RNA, 50 nM stem-looped RT primer, 1 × RT buffer, 0.25 mM each of dNTPs, 3.33 U/μl Multiscribe reverse transcriptase and 0.25 U/μl RNase Inhibitor. PCR amplification was carried out using sequence specific primers on the Applied Biosystems 7900 HT Fast Real-Time PCR system. The reactions were incubated in a 96-well optical plate at 95°C for 10 min, following by 40 cycles of 95°C for 15 s and 60°C for 10 min. Analysis of relative miRNA expression data was performed using ΔΔC_T _method with hsa-let-7a as an endogenous control [41]. Two negative controls were also used, ath-mir159a and cel-lin-4 as they show no expression in human tissue.
